# Covid-19 Confinement and Changes of Adolescent’s Dietary Trends in Italy, Spain, Chile, Colombia and Brazil

**DOI:** 10.3390/nu12061807

**Published:** 2020-06-17

**Authors:** María Belén Ruiz-Roso, Patricia de Carvalho Padilha, Diana C. Mantilla-Escalante, Natalia Ulloa, Paola Brun, Diofanor Acevedo-Correa, Wilza Arantes Ferreira Peres, Miquel Martorell, Mariana Tschoepke Aires, Letícia de Oliveira Cardoso, Fernanda Carrasco-Marín, Katherine Paternina-Sierra, Jhon E. Rodriguez-Meza, Piedad M. Montero, Giulia Bernabè, Anthony Pauletto, Xhoajda Taci, Francesco Visioli, Alberto Dávalos

**Affiliations:** 1 Laboratory of Epigenetics of Lipid Metabolism, Madrid Institute for Advanced Studies (IMDEA)-Food, CEI UAM + CSIC, 28049 Madrid, Spain; patricia@nutricao.ufrj.br (P.d.C.P.); diana.mantilla@imdea.org (D.C.M.-E.); 2 Instituto de Nutrição Josué de Castro, Universidade Federal do Rio de Janeiro, Rio de Janeiro 21941-902, Brazil; wilza@nutricao.ufrj.br; 3 Instituto de Puericultura e Pediatria Martagão Gesteira, Universidade Federal do Rio de Janeiro, Rio de Janeiro 21941-902, Brazil; marianataires@gmail.com; 4 Centro de Vida Saludable, Universidad de Concepción, Concepción 4070386, Biobío, Chile; nulloa@udec.cl (N.U.); mmartorell@udec.cl (M.M.); fercarrasco@udec.cl (F.C.-M.); 5 Departamento de Bioquímica Clínica e Inmunología, Facultad de Farmacia, Universidad de Concepción, Concepción 4070386, Chile; 6 Department of Molecular Medicine, University of Padova, I-35100 Padua, Italy; paola.brun.1@unipd.it (P.B.); giulia.bernabe@student.unife.it (G.B.); anthony.pauletto@phd.unipd.it (A.P.); xhoajda.taci@studenti.unipd.it (X.T.); francesco.visioli@unipd.it (F.V.); 7 Research Group in Innovation and Agricultural and Agroindustrial Development, University of Cartagena, Cartagena de Indias 48-152, Colombia; dacevedoc1@unicartagena.edu.co (D.A.-C.); paternina185@gmail.com (K.P.-S.); jrodriguezm3@unicartagena.edu.co (J.E.R.-M.); pmonteroc@unicartagena.edu.co (P.M.M.); 8 Departamento de Nutrición y Dietética, Facultad de Farmacia, Universidad de Concepción, Concepción 4070386, Chile; 9 Oswaldo Cruz Foundation, National School of Public Health, Rio de Janeiro 21041-210, Brazil; leticiadeoliveiracardoso@gmail.com; 10 Laboratory of Functional Foods, Madrid Institute for Advanced Studies Food (IMDEA Food), CEI UAM + CSIC, 28049 Madrid, Spain

**Keywords:** COVID-19, adolescents, diet, questionnaire, lifestyle, e-survey

## Abstract

Confinement due to the COVID-19 pandemic can influence dietary profiles, especially those of adolescents, who are highly susceptible to acquiring bad eating habits. Adolescents’ poor dietary habits increase their subsequent risk of degenerative diseases such as obesity, diabetes, cardiovascular pathologies, etc. Our aim was to study nutritional modifications during COVID-19 confinement in adolescents aged 10 to 19 years, compare them with their usual diet and dietary guidelines, and identify variables that may have influenced changes. Data were collected by an anonymous online questionnaire on food intake among 820 adolescents from Spain, Italy, Brazil, Colombia, and Chile. The results show that COVID-19 confinement did influence their dietary habits. In particular, we recorded modified consumption of fried food, sweet food, legumes, vegetables, and fruits. Moreover, gender, family members at home, watching TV during mealtime, country of residence, and maternal education were diversely correlated with adequate nutrition during COVID-19 confinement. Understanding the adolescents’ nutrition behavior during COVID-19 lockdown will help public health authorities reshape future policies on their nutritional recommendations, in preparation for future pandemics.

## 1. Introduction

On December 2019, an outbreak of pneumonia of then unknown etiology emerged in Wuhan City, Hubei Province in China, alerting the medical and scientific communities [1]. The causal agent was later identified in a new betacoronavirus called SARS-CoV-2, which can affect the lower respiratory tract and provoke bilateral pneumonia in humans [1]. This pathology—termed COVID-19 by the World Health Organization (WHO)—infected and killed thousands of people throughout the world; extraordinary measures have been taken in most countries, including Spain, Italy, Brazil, Chile, and Colombia. One of the containment measures was the total confinement of the population in their homes, also known as lockdown. This led to the disruption of most daily activities [2]. Different governments took different measures, yet all promulgated lockdown policies. On 9 March 2020, a national quarantine was imposed for Italy. A state of alarm and national lockdown was imposed on 14 March in Spain. A nationwide quarantine started in Colombia on 24 March 2020. On 27 March, Brazil announced a temporary ban on foreign air travelers and most state governors have imposed isolation policies. No national lockdown has been established in Chile, but some communities and urban areas did declare a mandatory quarantine at different times. However, on 16 March 2020, schools were closed in that country too (Supplementary Materials Table S1).

Confinement influences lifestyle, especially diet and physical activity. The World Health Organization and the Spanish Academy of Nutrition and Dietetics (2020) indicate that a healthy diet can help in the prevention and treatment of the disease [3]. Thus, recommendations have been published for food and nutrition during the period of confinement of the population, because there is a close relationship between the quality of a population’s food and its health [4]. Adequate nutrition is considered a potential factor for health in the early stages of life and adolescence [5]. At this stage, i.e., the transition period from childhood to adulthood, it is essential to acquire good eating behaviors that can concomitantly influence current health status and predisposition to diseases, e.g., obesity, diabetes, cardiovascular pathologies, etc., in adulthood. It is worth mentioning that the WHO implements and maintains health risk factor monitoring systems in adolescents [6].

It should be noted that, during confinement, it could become difficult to shop for fresh groceries and shortages of certain food products might happen. As recognized by The Food and Agriculture Organization (FAO), the COVID-19 pandemic has caused disruptions in food chains around the world, affecting both supply and demand [7]. Further, COVID-19 has made visible and magnified social inequalities, with the poorest families being the most affected ones [7].

On the other hand, there is the possibility that closer contact with family members and more home cooking due to COVID-19 confinement could teach adolescents skills that could improve their nutrition knowledge and behaviors, as several studies have indeed reported [8,9].

Therefore, in view of the current pandemic—when the population is suffering from social isolation—it is necessary to carry out research on the influence of this confinement on the quality of adolescents’ diet, considering some markers of healthy food intake. This could help public health authorities shape their recommendations, in terms of nutrition policies for adolescents, for future lockdown policies. Indeed, lifestyle lessons from the COVID-19 pandemic should prepare the whole population for the next one [10].

We aimed to assess the effects of COVID-19-induced confinement policies on self-reported nutritional habit modifications in adolescents from the five above-mentioned countries compared with their usual diet and with the dietary guidelines. Moreover, we aimed to identify potential variables that may have influenced this change.

## 2. Materials and Methods

### 2.1. Participants

This project was undertaken between 17 April 2020 and 25 May 2020. The target population was adolescents aged 10 to 19 years from several regions of Spain, Italy, Brazil, Colombia, and Chile (Supplementary Materials Table S2). The participants consented to participate in the study, with a digital informed consent form.

### 2.2. Study Design

This cross-sectional study used data collected via an anonymous online questionnaire consisting of more than 30 questions about dietary habits during COVID-19 confinement and the previous period. We distributed the questionnaire via social media, e.g., Twitter, WhatsApp or others (see below). In addition, researchers involved in this project distributed the survey to work colleagues.

Dietary practices were evaluated using a standardized adolescent questionnaire, the National School Health Survey–PeNSE; Pesquisa Nacional de Saúde do Escolar [11], which was slightly modified. Data collection was performed through a questionnaire divided into modules: sociodemographic and family features and dietary practices before and during confinement. The adolescent recorded the number of days on which they consumed the following foods or food groups during the week before confinement (BEFORE) and one week during confinement (COVID19): legumes; vegetables; fruit; sweet food; fried food (including packaged potatoes); processed meat (burger, sausage, mortadella, salami, ham, chicken nuggets, or sausages); sugar-sweetened beverages (SSB), and fast food. The PeNSE survey allows us to compare international indicators, especially those of the Global School-Based Student Health Survey [12], developed by the WHO, used in more than 90 countries around the world.

### 2.3. Data Collection

Data collection was carried out through a structured questionnaire created in Google Forms (Google LLC, Menlo Park, CA, USA). The questionnaire was divided into modules by subject: sociodemographic characteristics, dietary and lifestyle practices. The invitation to participate in the survey was made by social media (Facebook, Instagram and WhatsApp) or by e-mail to municipal authorities’ parents. The flow chart of participants of the study is depicted in Supplementary Figure S1.

### 2.4. Data Analysis

Initially, we compared the average intake of different food groups among the participants during COVID-19 confinement (COVID19) versus the previous period (BEFORE) by paired two-way Student’s *t*-test. The comparison of mean intake of different food groups during COVID-19 confinement classified by sociodemographic and family variables was assessed by two-way ANOVA. The independent categorical variables in Table of percentage of adolescents that maintain adequate food intake according to dietary guidelines by sociodemographic and familiar variables were assessed by chi-square test. A 95% confidence interval (95%CI) was adopted. To do this, two variables were created: a variable quantifying servings of legume, fruit, vegetables, fried food, sugary drinks, processed meat, and fast food intake per week, with seven categories (once, two, three, four, five, six and seven times per week) (this was the more important variable of this study); and a binary variable indicating if these adolescents met the WHO recommended diet during self-quarantine or longer home stays (yes/no). Socio-demographic variables collected were categorized as: gender: female and male; age: ≤14 years, 15–16 years and ≥17 years; number of people living at home: ≤3 people, 4–6 people and ≥7 people; watching TV during mealtimes: always, sometimes and never; and maternal education: none, primary, secondary, professional formation and complete university. A significance level of *p* < 0.05 was applied to all statistical analyses. GraphPad Prism 8 (version 8.3.0; Graph Pad Software Inc. San Diego, CA, USA) was used for all statistical analyses.

### 2.5. Ethical Approval

Ethical approval was obtained by the appropriate Ethical Committees of each country where the survey was performed. Specifically, by the IMDEA Food Research Ethics Committee in Spain (code IMD: PI-043); by the Ethical Committee of Human Inspired Technology Research Centre-Padova University in Italy (HIT Ethical Committee 33035 22 April 2020); by the Research Ethics Committee of IPPMG–Federal University of Rio de Janeiro in Brazil (approval number: 3,975,744); by the Cartagena committee and the University of Cartagena in Colombia (acta N° 134) and by the University of Concepción School of Medicine Bioethical Committee in Chile (CEBB 646-2020). The study is in accordance with the ethical principles of non-maleficence, beneficence, justice and autonomy, contained in the ethical resolutions of each country, according to the Helsinki declaration. An informed consent form was signed digitally by one of the participants’ guardians before initiating the survey.

## 3. Results and Discussion

### 3.1. Socio-Demographic Characteristics

A total of 820 adolescents from several regions of Spain, Italy, Brazil, Colombia, and Chile participated in this study (Supplementary Materials Table S2). The average age of adolescents was 15 years (18.7%), with more girls (61.1%) than boys (38.9%). As for maternal education, 43.3% reported that their mothers did not have a university degree. The sample was about equally divided between the five countries. All sociodemographic variables are included in Table 1.

### 3.2. Confinement Due to COVID-19 Changed Dietary Trends among Adolescents

**Figure 1:**
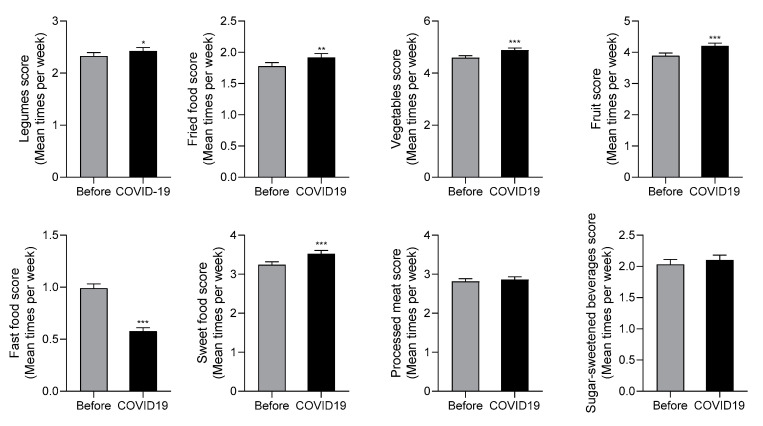
Comparison of average dietary intake among adolescents during COVID-19 confinement (COVID19) and the previous period (Before). Data are means ± SEM. Comparison between groups by paired two-way Student’s *t*-test. * *p* < 0.05, ** *p* < 0.001, *** *p* < 0.0001. *N* = 820.

**Figure 2:**
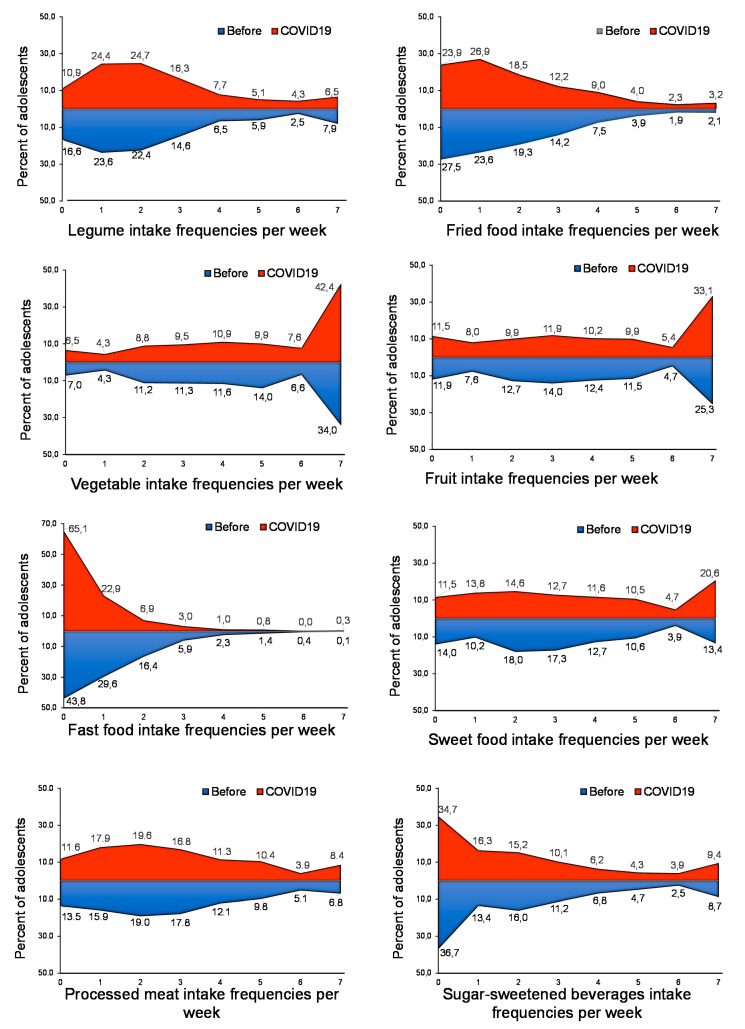
Food intake frequencies to compare dietary patterns during COVID-19 confinement (COVID19, red area) and the previous period (BEFORE, blue area), expressed by percent of adolescents according to weekly intake frequency of each food group. *N* = 820.

Figure 1 reports the mean food score per week during and before the COVID-19 period. Legumes, vegetables, and fruit intakes were significantly increased during COVID-19 confinement. In addition, the distribution intake frequencies show an increase in the number of adolescents who consume the recommended weekly servings of legumes during confinement (2, 3, or 4 servings per week; from 22.7, 15.4 and 6.1% before to 25.0%, 16.0% and 7.4% during COVID-19 confinement) (Figure 2).

It is also important to highlight the changes in the pattern of vegetable and fruit consumption of the adolescents of this survey. Forty-three percent of adolescents consumed vegetables every day during confinement versus 35.2% who did it before (Figure 2). Similarly, only 25.5% of adolescents surveyed consumed at least one piece of fruit per day before COVID-19 versus 33.2% during confinement (Figure 2). These results are not surprising because the sale of this type of food has increased since the beginning of confinement [13] and the population has more time to cook at home. Further, the WHO recommends legumes, fruits and vegetables as the best food items during self-quarantine or longer home stays [14]. 

In addition, we report that fast food intake was dramatically reduced in adolescents during COVID-19 confinement (Figure 1). While before confinement only 44.6% of adolescents consumed fast food less than once a week, this figure increased to 64% during confinement (Figure 2). It is possible that home cooking could reduce the incidence of chronic diseases [15], but any long-term improvements caused by increased cooking might be small compared to the more problematic and enduring effects of this crisis on children and adolescents [16,17].

By contrast, fried and sweet food average intakes increased significantly during COVID-19 confinement (Figure 1). Fourteen percent of adolescents consumed sweet food every day before COVID-19, which increased to 20.7% during confinement (Figure 2). Similarly, Figure 2 shows the increase of adolescents who consumed fried foods 4–7 days per week, from 7.4%, 3.7%, 1.8% and 2.1% before to 8.8%, 3.8%, 2.2% and 2.9% during confinement. These results confirm previous studies that suggested that the confinement could lead to irregular eating patterns and frequent snacking in adolescents due to boredom and stress [17,18]. It is also important to highlight that these dietary habits are associated with a higher caloric intake and an increased risk of obesity [19].

There were no changes in self-reported processed meat and sugar-sweetened beverage intakes (SSB) in these populations during COVID-19 isolation (Figure 1 and Figure 2). In contrast, in a recent study of Italian adults, purchases of ready-made meals were reduced during the lockdown [20].

### 3.3. Adolescents’ Dietary Habits during COVID-19 Confinement According to Sociodemographic and Family Characteristics and the Modifications Versus the Previous Period

Table 2 shows the changes in adolescents’ dietary trends classified by sociodemographic and family characteristics due to COVID-19 confinement. Only the most notable results will be discussed. The highest rates of adherence to the weekly food intake recommendation were in females among adolescents living in Europe and those who reported a higher maternal education (Table 3).

#### 3.3.1. Gender

**Figure 3:**
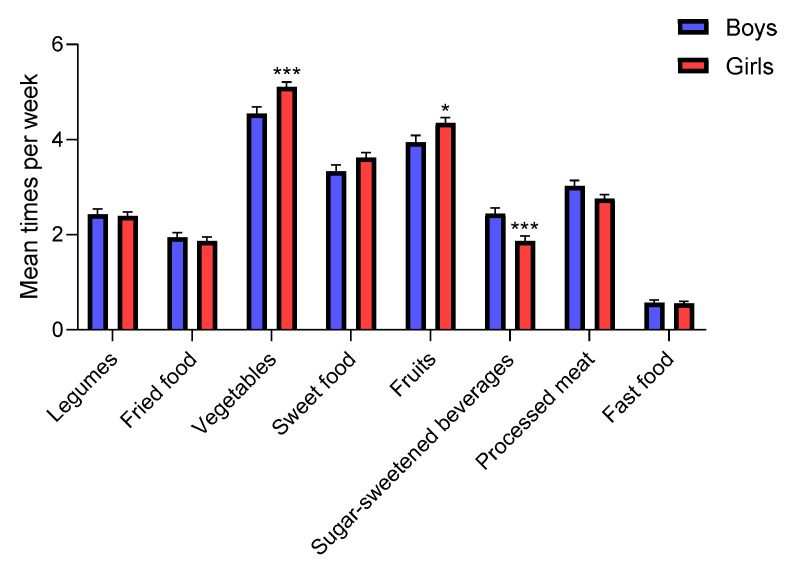
Comparison of average dietary intake among adolescents during COVID-19 confinement by gender. Data are means ± SEM. Comparison between groups by paired two-way ANOVA. * *p* < 0.05, *** *p* < 0.0001.

The gender classification results show that females significantly increased their vegetable (from 4.8 before to 5.1 times per week, *p* < 0.0001) and fruit intake (from 4.0 before to 4.4 times per week, *p* < 0.0001) during confinement (Table 2). On the other hand, males also showed an increase in vegetable consumption (from 4.0 before to 4.4 times per week during COVID-19, *p* = 0.0007), and processed meat intake (from 2.9 before to 3.0 time per week during COVID-19, *p* = 0.0182) but did not change their average fruit consumption (Table 2). Moreover, when comparing the average food intake by gender, we confirm that girls consumed significantly more fruits and vegetables during COVID-19 confinement and fewer SSB than boys, in our survey (Figure 3). These results are in concordance with previous observational studies about nutritional behaviors and differences between sexes [21]. 

The potential correlates of adequate nutrition with sociodemographic and family variables are shown in Table 3. The most evident differences between girls and boys (in terms of dietary guidelines) were found for SSB (*p* < 0.0001). Only 42.9% of males versus almost 57.2% of females occasionally drank SSB, adhering to the recommendations of limiting the use of such beverages [22] (Table 3). This result is very relevant because SSB and sweet food increase the risk of overweight and obesity, type 2 diabetes mellitus, cardiovascular disease, among others noxious health effects [22].

#### 3.3.2. Age

Overweight and obesity in childhood lead to a high risk of these conditions in adolescence and adulthood, and earlier-onset obesity is associated with a greater risk of adult overweight or obesity [23]. Therefore, it is important to evaluate changes in dietary patterns by age due to confinement to allow one to identify young people at risk of nutritional inadequacies and the development of eating disorders.

**Figure 4:**
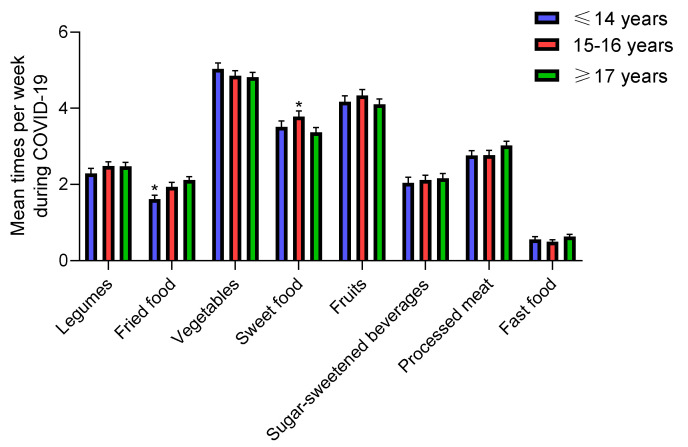
Mean dietary intake among adolescents during COVID-19 confinement by age. Data are means ± SEM. Comparison between groups by paired two-way ANOVA.

Only adolescents over the age of 14 significantly increased vegetable and fruit intake during COVID-19 confinement versus before confinement (Table 2). Adolescents under the age of 14 significantly increased the average consumption of fried and sweet foods (from 1.3 and 3.2 before to 1.6 and 3.5 during the COVID-19 pandemic, respectively; *p* = 0.0025 and *p* = 0.0386) (Table 2). It is important to note that we recorded a dramatic increase in sweet food consumption in those over 17 years of age due to COVID-19 confinement (*p* = 0.001), but all teens, regardless of age, consumed sweets 3 to 4 times per week. There were no differences in adolescents’ mean food intake during COVID-19 confinement by age (Figure 4), but we found significant differences by age in legume, fried food, vegetable and fast food average intake during confinement compared with the recommended dietary allowance (legumes: 3 or 4 servings per week [24], fried food: occasional [14], vegetables: diary [25] and fast food: occasional [26,27,28] (Table 3). However, in relation to age, no significant association with dietary patterns was identified, possibly because the sample consisted only of adolescents and with a low range of age variation (10 to 19 years).

#### 3.3.3. Country

**Figure 5:**
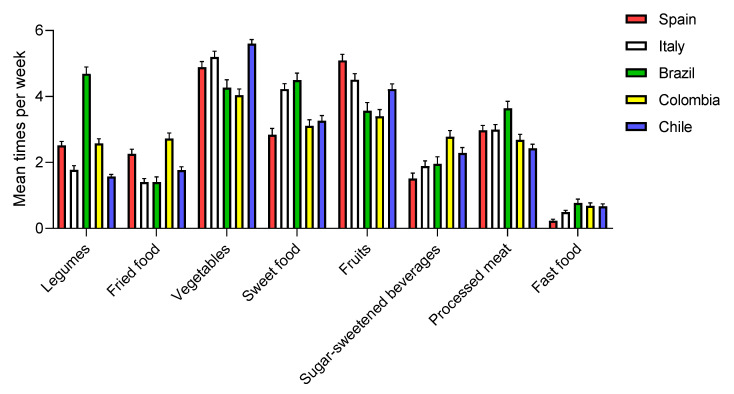
Mean dietary intake among adolescents during COVID-19 confinement by country. Data are means ± SEM.

The country of residence is strongly related to mean food intake during the COVID-19 pandemic (Figure 5) and to the modification of dietary trend among adolescents in this period versus habitual diet (Table 2). Many factors, such as socio-economic status, fad diets, religion, and traditions of each country, influence dietary trends [29,30].

Brazilian adolescents had a higher average legume intake (Figure 5) versus the other countries. In addition, all adolescents except the Spanish one significantly increased the consumption of legumes during confinement (Table 2).

Adolescents from Spain, Brazil, and Chile, but not Italy and Colombia, increased vegetable consumption during confinement (Table 2). In addition, the Colombian adolescents had low overall fruit and vegetable consumption, despite having increased their fruit intake in a significant way during confinement. These results are consistent with previous studies that show that the Colombian population does not consume the recommended amount of fruits and vegetables [31]. In our study, Spain and Italy were the countries with the greatest mean consumption of fruits (4–5 times per week), and significantly increased their consumption during COVID-19 confinement (Table 2). A total of 49.3% of Spanish adolescents met the fruit intake recommendations versus the almost 23% of Colombian subjects (Table 3).

Colombian adolescents significantly decreased sugar-sweetened beverage intakes during the COVID-19 pandemic, but this country remained the biggest consumer of SSB as compared with Spain, Italy, Brazil, and Chile (Table 2). Only 36.0% of Colombian adolescents drank SBB only occasionally during the COVID-19 lockdown, versus 64.5% of Spanish adolescents (Table 3).

Chileans significantly increased fried food intake during COVID-19 (*p* < 0.0001), but again Colombia was the biggest overall consumer of fried food (almost three times per week versus 1.4 to 2.3 times per week for the other countries (Table 2)). It should be noted that all adolescents decreased their weekly fast food consumption and all countries showed the same average fast food intake during confinement (0.2–0.8 times per week) (Figure 5 and Table 2). Detailed statistical analysis of Figure 5 is described in Table 4.

These results are especially relevant because experiences from previous epidemics have shown that there is a need to maintain optimal nutrition at individual and global levels, in order to improve the physical and mental health of the population [32]. In this sense, knowing the dietary habits in each country is necessary to encourage a healthy lifestyle after COVID-19 confinement or the development of future reactions to unavoidable pandemics.

#### 3.3.4. Maternal Education

**Figure 6:**
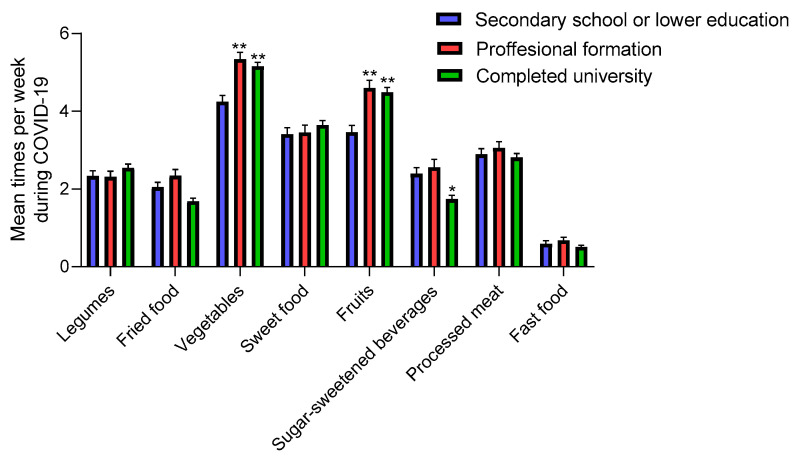
Mean dietary intake among adolescents during COVID-19 confinement by maternal education. Data are means ± SEM. Comparison between groups by paired two-way ANOVA. * *p* < 0.001, ** *p* < 0.0001 compared to secondary school or lower education group.

Adolescents whose mothers had education levels higher than secondary school level significantly increased their consumption of fruits and vegetables during confinement (Table 2), and were the ones who consumed the most fruits and vegetables during and before compared to the rest of the adolescents (Figure 6). By contrast, adolescents who reported high maternal education also significantly increased their consumption of sweet food (Table 2). In spite of this, there were no significant differences in mean sweet food intake between them (Figure 6). It is important to highlight the low consumption of SSB among adolescents whose mother had a university degree versus other groups (*p* < 0.001) during COVID-19 (Figure 5). Our results suggest that there was greater adherence to the unhealthy eating pattern during COVID-19 among adolescents whose mothers had low education. Whether this is related to the family income is not known and deserves further investigation.

#### 3.3.5. Number of Family Members at Home

**Figure 7:**
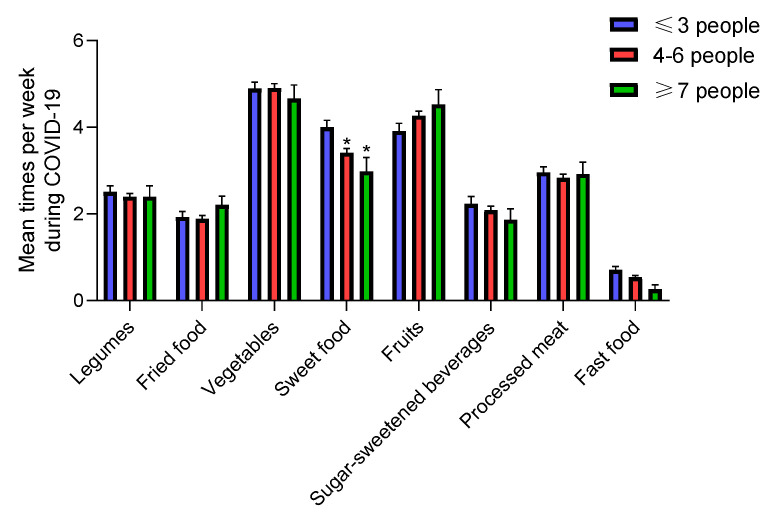
Mean dietary intake among adolescents during COVID-19 confinement by family members. Data are means ± SEM. Comparison between groups by paired two-way ANOVA. * *p* < 0.001 compared to the ≤3 people group.

There were differences in adolescents’ sweet food intake during COVID-19 confinement as stratified by family members. The ≤3 people group had a higher average sweet food intake (Figure 7) versus the other groups (*p* < 0.001), and significantly increased their consumption during COVID-19 confinement, unlike the other groups (Table 2). Furthermore, only 3.4% of adolescents of ≤3 people group and 2.6% of ≥7 people group ate only occasionally sweet food versus the 19% of 4–5 people group (*p* < 0.001) (Table 3). It is important to highlight that the lowest rates of adherence to the weekly vegetables, sweet food and fast food intake recommendation were in those adolescents who belong to the ≥7 people group (Table 3).This could be because larger families reduce the amount of resources, such as time, energy, or money available to each child [33]. However, we cannot prove beyond reasonable doubt that larger family size influences diet quality, either directly or indirectly.

#### 3.3.6. Watching TV during Mealtimes

**Figure 8:**
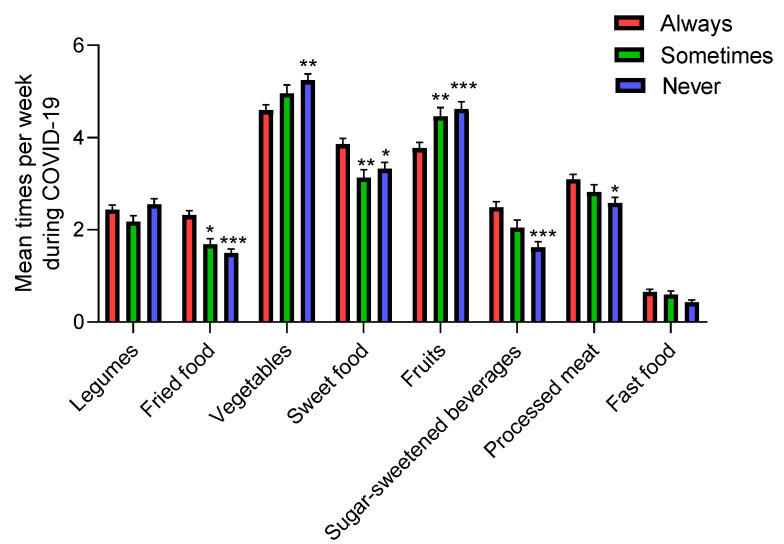
Mean dietary intake among adolescents during COVID-19 confinement by viewing TV during mealtimes. Data are means ± SEM. Comparison between groups by paired two-way ANOVA. * *p* < 0.05, ** *p* < 0.001, *** *p* < 0.0001 compared to the Always group.

The results show that TV viewing during mealtimes is related to lower consumption of vegetables and fruits during the COVID-19 period and a higher fried food, sweet food, and SSB consumption (Figure 8). Further, those adolescents who always watched TV during mealtimes significantly increased their fried and sweet food intake during COVID-19 confinement versus before, and those adolescents who never watched TV during mealtimes significantly increased their vegetable and fruit intake (Table 2). We found significant differences in vegetable, fruit, fried food, sweet food, and SSB intake during confinement compared with the recommended dietary allowance in those adolescents who always watch TV during mealtimes versus the other two groups, proving that watching TV during mealtimes was associated with poorer dietary quality among adolescents (Table 3). 

As a limitation of this study, we did not investigate modifications of adolescent’s dietary trends as correlated to economic status, daily food intake times, and amount and availability of food in each region or country. We also do not have data regarding sleep, physical activity, and time spent TV watching. 

## 4. Conclusions

In conclusion, our findings provide the first description of how the COVID-19 pandemic has modified dietary trends of adolescents from Spain, Italy, Brazil, Colombia, and Chile. These new habits could be acquired and have some later impact on health. Due to confinement, it appears that families had more time to cook and improve eating habits by increasing legume, fruit, and vegetable intake, even though this, apparently, did not increase the overall diet quality. Further, adolescents also exhibited a higher sweet food consumption, likely due to boredom and stress produced by COVID-19 confinement. This study shows the association between gender, country of residence, family members at home, watching TV during mealtimes and maternal education variables with adequate nutrition during COVID-19 confinement. Therefore, it is important to generate future large-scale studies that analyze eating habits to encourage the adoption of healthy diets among adolescents, especially after this period of confinement. Understanding the present adolescent’s nutrition behavior during Covid-19 lockdown will help public health authorities reshape future policies on adolescents’ nutritional recommendations, when new pandemics arrive and lockdown policies are implemented.

## Figures and Tables

**Table 1:** Socio-demographic characteristics of the 820adolescents who filled out the questionnaire.

Variables	Sample%	Variables	Sample%
**Gender (*N* = 810)**		**Maternal education (*N* = 779)**	
Boys	38.89	None	1.54
Girls	61.11	Primary	10.14
**Age (years) (*N* = 820)**		Secondary	15.92
≤14	27.68	Professional formation	17.97
15–16	30.98	Complete university	54.43
≥17	41.34	**Family members (*N* = 820)**	
**Country (*N* = 820)**		≤3	25.73
Spain	18.54	4–6	67.80
Brazil	14.02	≥7	6.46
Colombia	19.63	**Viewing TV during mealtimes (*N* = 820)**	
Chile	26.22	Always	46.71
Italy	21.59	Sometimes	19.88
		Never	33.41

**Table 2:** Comparison of adolescents’ dietary trends before and during COVID-19 confinement classified by sociodemographic and familiar characteristics.

Variables	Legumes			Fried Food			Vegetables			Sweet Food			Fruits			Sugar-Sweetened Beverages			Processed Meat			Fast Food		
	Mean	95% IC	*p*-Value	Mean	95% IC	*p*-Value	Mean	95% IC	*p*-Value	Mean	95% IC	*p*-Value	Mean	95% IC	*p*-Value	Mean	95% IC	*p*-Value	Mean	95% IC	*p*-Value	Mean	95% IC	*p*-Value
**Gender (*N* = 810)**																								
Male	2.3–2.4	(2.1–2.6)–(2.2–2.6)	0.1223	1.8–1.9	(1.6–2.0)–(1.8–2.1)	0.0803	4.3–4.6	(4.1–4.6)–(4.3–4.8)	0.0028 **	3.1–3.3	(2.8–3.3)–(3.0–3.6)	0.0174 *	3.7–3.9	(3.5–4.0)–(3.7–4.2)	0.0556	2.3–2.4	(2.1–2.7)–(2.0–2.6)	0.193	2.9–3.0	(2.6–3.1)–(2.8–3.3)	0.0182 *	0.9–0.6	(0.8–1.0)–(0.5–0.7)	<0.0001 ***
Female	2.3–2.4	(2.1–2.5)–(2.3–2.6)	0.0768	1.7–1.9	(1.6–1.8)–(1.7–2.0)	0.0224 *	4.8–5.1	(4.6–5.0)–(4.9–5.3)	<0.0001 ***	3.3–3.6	(3.2–3.5)–(3.4–3.8)	0.0067 **	4.0–4.4	(3.7–4.2)–(4.1–4.6)	<0.0001 ***	1.9–1.9	(1.7–2.1)–(1.7–2.1)	0.9232	2.8–2.8	(2.6–3.0)–(2.6–2.9)	0.3263	1.0–0.6	(0.9–1.1)–(0.5–0.6)	<0.0001 ***
**Age (years) (*N* = 820)**																								
≤14	2.3–2.3	(2.0–2.6) – (2.0–2.6)	>0.9999	1.3–1.6	(1.1–1.5)–(1.4–1.8)	0.0025 **	4.9–5.0	(4.5–5.2)–(4.7–5.3)	0.0707	3.2–3.5	(2.9–3.3)–(3.1–3.8)	0.0386 *	4.1–4.2	(3.8–4.4)–(3.9–4.5)	0.4900	1.8–2.0	(1.5–2.1)–(1.7–2.3)	0.0518	2.6–2.8	(2.4–2.9)–(2.5–3.0)	0.2457	0.8–0.6	(0.6–0.9)–(0.4–0.7)	0.0056 **
15–16	2.4–2.5	(2.1–2.6) – (2.3–2.7)	0.0954	1.9–1.9	(1.7–2.2)–(1.7–2.2)	0.9258	4.4–4.9	(4.1–4.7)–(4.6–5.1)	<0.0001 ***	3.6–3.8	(3.4–3.9)–(3.5–4.1)	0.2576	4.1–4.3	(3.8–4.4)–(4.0–4.7)	0.0139 *	2.2–2.1	(1.9–2.5)–(1.8–2.4)	0.469	2.7–2.8	(2.5–3.0)–(2.5–3.0)	0.8028	1.0–0.5	(0.9–1.2)–(0.4–0.6)	<0.0001 ***
≥17	2.4–2.5	(2.1–2.6) – (2.3–2.7)	0.0237 *	2.0–2.1	(1.8–2.1)–(1.9–2.3)	0.0448 *	4.6–4.8	(4.3–4.8)–(4.6–5.1)	0.002 **	3.0–3.4	(2.8–3.2)–(3.2–3.6)	0.001 **	3.6–4.1	(3.8–3.9)–(3.8–4.4)	<0.0001 ***	2.1–2.2	(1.9–2.3)–(1.9–2.4)	0.5048	3.0–3.0	(2.8–3.2)–(2.8–3.3)	0.9426	1.1–0.6	(0.9–1.2)–(0.6–0.7)	<0.0001 ***
**Country (*N* = 820)**																								
Spain	2.5–2.5	(2.3–2.7) – (2.3–2.8)	0.9375	2.2–2.3	(2.0–2.5)–(2.0–2.5)	0.7728	4.5–4.9	(4.1–4.8)–(4.6–5.2)	<0.0001 ***	2.7–3.1	(2.4–3.0)–(2.8–3.5)	0.0075 **	4.5–5.1	(4.1–4.9)–(4.7–5.5)	0.0008 ***	1.4–1.5	(1.1–1.6)–(1.2–1.8)	0.2383	2.7–3.0	(2.4–3.0)–(2.7–3.3)	0.0534	0.8–0.2	(0.7–1.0)–(0.1–0.3)	<0.0001 ***
Italy	1.6–1.8	(1.4–1.8) – (1.5–2.0)	0.0308 *	1.2–1.4	(1.0–1.4)–(1.2–1.6)	0.0375 *	4.5–4.7	(4.2–4.9)–(4.4–5.1)	0.0723	3.7–4.2	(3.4–4.0)–(3.9–4.5)	<0.0001 ***	4.2–4.5	(3.9–4.6)–(4.2–4.9)	0.0172 *	1.8–1.9	(1.4–2.0)–(1.6–2.2)	0.1321	3.0–3.0	(2.7–3.3)–(2.7–3.3)	0.6935	0.7–0.5	(0.6–0.9)–(0.4–0.6)	0.0114 *
Brazil	5.1–4.7	(4.7–5.5) – (4.3–5.1)	0.0031 **	1.6–1.7	(1.4–1.7)–(1.5–1.9)	0.1613	4.4–4.6	(4.1–4.6)–(4.3–4.9)	0.0039 **	3.1–4.0	(2.9–3.4)–(3.7–4.3)	<0.0001 ***	3.6–3.9	(3.3–3.9)–(3.6–4.2)	0.0097 **	1.6–2.0	(1.2–1.9)–(1.5–2.4)	0.03 *	3.2–3.3	(3.0–3.4)–(3.0–3.5)	0.3427	1.4–0.8	(1.2–1.6)–(0.5–1.0)	<0.0001 ***
Colombia	2.2–2.6	(2.0–2.5)–(2.3–2.9)	0.0026 **	2.7–2.7	(2.4–3.0)–(2.4–3.1)	0.7284	3.9–4.0	(3.6–4.3)–(3.7–4.4)	0.4793	3.4–3.4	(3.1–3.6)–(3.2–3.7)	0.7301	3.5–3.8	(3.2–3.7)–(3.5–4.1)	0.0195 *	3.0–2.5	(2.6–3.3)–(2.2–2.8)	0.0008 ***	3.0–3.0	(2.7–3.2)–(2.7–3.1)	0.4084	1.3–0.7	(1.1–1.4)–(0.5–0.8)	<0.0001 ***
Chile	1.4–1.6	(1.3–1.5)–(1.4–1.7)	0.0021	1.4–1.8	(1.2–1.6)–(1.6–2.0)	<0.0001 ***	4.9–5.2	(4.6–5.1)–(4.9–5.4)	0.0019 **	3.0–3.3	(2.7–3.2)–(3.0–3.6)	0.0381 *	4.1–4.2	(3.7–4.4)–(3.9–4.5)	0.2454	2.0–2.3	(1.7–2.3)–(1.9–2.6)	0.0598	2.6–2.6	(2.4–2.9)–(2.4–2.9)	0.5145	0.8–0.7	(0.6–1.0)–(0.5–0.8)	0.0392 *
**Maternal education (*N* = 779)**																								
None	3.0–3.3	(1.7–4.9)–(1.5–4.5)	0.3388	1.8–2.2	(1.2–3.1)–(0.5–3.2)	0.6326	2.9–3.3	(2.1–4.6)–(1.6–4.3)	0.2098	3.4–4.2	(2.8–3.9)–(3.6–4.8)	0.0143 *	3.3–3.6	(1.7–4.9)–(2.1–5.1)	0.6332	3.1–3.8	(2.0–5.6)–(1.2–5.0)	0.3625	2.9–3.3	(1.6–4.3)–(1.8–4.9)	0.4699	1.6–1.8	(0.8–2.4)–(0.2–3.3)	0.7126
Primary	2.0–2.2	(1.6–2.4)–(1.8–2.6)	0.3204	2.0–2.0	(1.7–2.3)–(1.7–2.4)	0.754	4.5–4.9	(4.1–5.0)–(4.4–5.4)	0.0883	3.2–3.2	(2.7–3.7)–(2.7–3.7)	0.8764	3.6–3.9	(3.0–4.1)–(3.4–4.5)	0.0575	1.9–2.0	(1.5–2.4)–(1.5–2.5)	0.8761	2.8–2.7	(2.4–3.2)–(2.3–3.1)	0.6177	1.0–0.5	(0.7–1.2)–(0.3–0.8)	0.0007 ***
Secondary	2.1–2.2	(1.–2.4)–(1.8–2.6)	0.3204	1.9–2.1	(1.6–2.2)–(1.7–2.4)	0.2244	3.8–4.0	(3.4–4.3)–(3.5–4.4)	0.2896	3.5–3.5	(3.1–3.9)–(3.0–3.9)	0.9364	2.9–3.1	(2.5–3.3)–(2.7–3.5)	0.352	2.5–2.6	(2.1–2.9)–(2.2–3.0)	0.7131	2.9–3.1	(2.8–3.5)–(2.6–3.4)	0.226	1.0–0.5	(0.8–1.2)–(0.4–0.7)	<0.0001 ***
Professional formation	2.0–2.3	(1.7–2.3)–(2.0–2.6)	0.004**	1.9–2.3	(1.7–2.3)–(2.1–2.7)	0.0025**	4.8–5.3	(4.4–5.2)–(5.0–5.7)	0.0006***	3.1–3.5	(2.7–3.4)–(3.1–3.8)	0.0321 *	4.0–4.6	(3.6–4.4)–(4.2–5.0)	0.0002 ***	2.4–2.6	(2.0–2.8)–(2.2–3.0)	0.4696	2.8–3.1	(2.5–3.2)–(2.7–3.4)	0.1009	1.0–0.7	(0.8–1.2)–(0.5–0.8)	0.0006 ***
Complete univesity	2.5–2.5	(2.3–2.7)–(2.3–2.7)	0.7896	1.6–1.7	(1.5–1.8)–(1.5–1.8)	0.3039	4.8–5.2	(4.6–5.1)–(5.0–5.4)	<0.0001***	3.2–3.6	(3.0–3.4)–(3.4–3.9)	<0.0001 ***	4.2–4.5	(4.0–4.5)–(4.3–4.8)	0.0054 **	1.6–1.7	(1.4–1.8)–(1.5–1.9)	0.1633	2.8–2.8	(2.6–3.0)–(2.6–3.0)	0.7943	0.9–0.5	(0.8–1.0)–(0.4–0.6)	<0.0001 ***
**Family members (*N* = 820)**																								
≤3	2.3–2.5	(2.1–2.7)–(2.2–2.8)	0.0826	1.7–1.9	(1.5–2.0)–(1.7–2.2)	0.0326 *	4.6–4.9	(4.3–4.9)–(4.6–5.2)	0.0042 **	3.3–4.0	(3.0–3.6)–(3.7–4.3)	<0.0001 ***	3.7–4.0	(3.4–4.1)–(3.6–4.3)	0.0808	2.1–2.2	(1.8–2.4)–(1.9–2.6)	0.2819	2.9–3.0	(2.6–3.1)–(2.7–3.2)	0.356	1.2–0.7	(1.0–1.3)–(0.5–0.9)	<0.0001 ***
4–6	2.3–2.4	(2.2–2.5)–(2.3–2.6)	0.1148	1.8–1.9	(1.6–1.9)–(1.7–2.0)	0.0354*	4.6–4.9	(4.5–4.8)–(4.7–5.1)	<0.0001 ***	3.3–3.4	(3.0–3.4)–(3.2–3.6)	0.0926	4.0–4.3	(3.8–4.2)–(4.0–4.5)	0.0018 **	2.1–2.1	(1.9–2.2)–(1.9–2.3)	0.6841	2.8–2.8	(2.6–3.0)–(2.6–3.0)	0.7992	0.9–0.5	(0.8–1.1)–(0.5–0.6)	<0.0001 ***
≥7	2.3–2.4	(1.8–2.8)–(1.9–3.0)	0.308	2.2–2.2	(1.7–2.6)–(1.8–2.6)	0.9168	4.1–4.5	(3.6–4.6)–(4.0–5.0)	0.0045 **	3.0–3.0	(2.3–3.6)–(2.3–3.7)	0.9234	3.4–4.5	(2.8–4.0)–(3.9–5.2)	0.0017 **	1.7–1.9	(1.2–2.2)–(1.4–2.4)	0.499	2.8–2.9	(2.3–3.3)–(2.4–3.5)	0.6483	0.1–0.3	(0.6–1.2)–(0.1–0.5)	<0.0001 ***
**TV during mealtimes (*N* = 820)**																								
Always	2.4–2.4	(2.2–2.6)–(2.3–2.6)	0.3225	2.0–2.3	(1.9–2.2)–(2.1–2.5)	0.0003 ***	4.4–4.6	(4.2–4.6)–(4.4–4.8)	0.0208 *	3.4–3.9	(3.1–3.6)–(3.6–4.1)	<0.0001 ***	3.5–3.8	(3.3–3.8)–(3.5–4.0)	0.025 *	2.5–2.5	(2.2–2.7)–(2.2–2.7)	0.7866	3.0–3.1	(2.8–3.2)–(2.9–3.3)	0.2836	1.0–0.7	(0.9–1.1)–(0.6–0.8)	<0.0001 ***
Sometimes	2.0–2.2	(1.7–2.3)–(2.0–2.4)	0.067	1.6–1.7	(1.3–1.8)–(1.4–1.9)	0.2683	4.7–5.0	(4.3–5.0)–(4.6–5.3)	0.0488 *	3.3–3.1	(2.9–3.6)–(2.8–3.5)	0.3887	3.8–4.3	(3.8–4.6)–(4.1–4.8)	0.0631	2.0–2.0	(1.6–2.3)–(1.7–2.4)	0.43	2.7–2.8	(2.4–3.0)–(2.5–3.1)	0.3175	1.0–0.6	(0.8–1.2)–(0.4–0.8)	0.0004 ***
Never	2.4–2.5	(2.2–2.7)–(2.3–2.8)	0.1096	1.5–1.5	(1.4–1.7)–(1.3–1.7)	0.5086	4.8–5.2	(4.5–5.0)–(5.0–5.5)	<0.0001 ***	3.1–3.3	(2.8–3.3)–(3.0–3.6)	0.0459 *	4.2–4.6	(3.9–4.5)–(4.3–4.9)	0.0005 ***	1.5–1.6	(1.2–1.7)–(1.3–1.9)	0.3032	2.6–2.6	(2.4–2.9)–(2.3–3.8)	0.4801	0.9–0.4	(0.7–1.1)–(0.3–0.5)	<0.0001 ***

Paired two-way Student’s *t*-test. Data are shown as mean of food intakes and respective 95% confidence intervals (95%CI). * *p* < 0.05, ** *p* < 0.001, *** *p* < 0.0001. *N* = 820.

**Table 3:** Percentage of adolescents that maintain adequate food intake according to dietary guidelines by sociodemographic and familiar variables.

Variables	Legumes			Fried Food			Vegetables			Sweet Food			Fruits			Sugar-Sweetened Beverages			Processed Meat			Fast Food		
	%	95% IC	*p*-Value	%	95% IC	*p*-Value	%	95% IC	*p*-Value	%	95% IC	*p*-Value	%	95% IC	*p*-Value	%	95% IC	*p*-Value	%	95% IC	*p*-Value	%	95% IC	*p*-Value
**Gender (*N* = 810)**			0.241			0.4004			0.0109*			0.0277 *			0.4586			<0.0001 ***			0.4586 *			0.2628
Boys	21.3	18.5–24.0		48.9	45.5–52.3		37.8	34.5–41.1		29.5	26.4–32.0		31.4	28.3–34.6		42.9	39.5–46.2		25.1	22.4–28.6		86.0	83.7–88.4	
Girls	24.8	21.9–27.8		51.9	48.5–55.3		46.9	43.5–50.3		22.6	19.8–25.5		33.9	30.8–37.1		57.2	53.8–60.6		32.3	29.1–36.0		88.7	86.5–90.9	
**Age (years) (*N* = 820)**			0.0063 **			0.0006 ***			0.0585			0.3118			0.3931			0.4846			0.523			0.013 *
≤14	15.86	1.3.4–18.3		58.59	55.2–62.2		49.78	46.4–53.2		27.31	24.3–30.4		29.96	36.8–33.1		54.63	51.2–58.0		28.19	25.1–31.0		88.55	86.4–91.7	
15–16	25.59	22.6–28.6		53.15	49.7–56.7		41.34	38.0–44.7		21.65	18.8–24.5		35.83	32.5–39.1		49.21	45.8–52.6		31.89	28.7–35.0		90.94	89.0–92.9	
≥17	26.84	23.8–29.9		42.77	39.4–46.1		40.12	36.8–43.5		25.96	23.0–29.0		33.04	29.8–36.2		51.03	47.6–54.4		28.61	25.5–32.0		84.37	81.9–86.9	
**Country (*N* = 820)**			<0.0001 ***			<0.0001 ***			<0.0001 ***			0.0006 **			<0.0001 ***			0.0002 **			0.0219 *			0.0012 *
Spain	34.87	31.6–38.1		42.11	38.7–45.5		33.55	30.1–36.8		38.82	35.5–42.2		49.34	45.9–52.8		64.47	61.2–67.8		25.66	22.7–29.0		98.03	97.1–99.0	
Brazil	24.35	21.4–27.3		63.48	60.2–67.8		35.65	32.4–38.9		13.04	10.7–15.3		23.48	20.6–26.4		53.04	49.6–56.5		20.00	17.3–23.0		81.74	79.1–84.4	
Colombia	30.43	27.3–33.6		34.78	31.5–38.0		29.81	26.7–32.9		31.06	27.9–34.2		22.98	20.1–25.9		36.02	32.7–39.3		37.27	34.0–41.6		81.99	79.4–846.6	
Chile	10.70	8.6–12.8		52.09	48.7–55.5		55.814	52.4–59.2		26.51	23.5–29.5		28.37	25.3–31.5		51.16	47.7–54.6		33.49	30.3–37.6		85.58	83.2–88.0	
Italy	22.03	19.2–24.9		61.02	57.7–64.4		53.11	49.7–56.5		13.56	11.2–15.9		40.11	36.8–42.5		53.67	50.3–57.1		27.12	24.1–30.2		89.83	87.8–91.9	
**Maternal education (*N* = 779)**			0.6915			0.0103*			<0.0001 ***			0.9074			0.0015 **			0.0007 ***			0.8702			0.0427 *
None	25.00	22.0–28.0		66.67	63.4–70.0		8.33	6.4–10.3		25.00	22.0–28.0		16.67	14.0–19.3		41.67	38.2–45.1		25.00	22.0–28.0		66.67	63.4–70.0	
Primary	25.32	22.3–28.4		45.57	42.1–49.1		40.51	37.1–44.0		27.85	24.7–31.0		26.58	23.5–29.7		51.90	48.4–55.4		29.11	25.9–32.3		92.41	90.5–94.3	
Secondary	18.548	15.8–21.3		54.03	50.5–57.5		33.06	29.8–36.4		26.61	23.5–29.7		20.97	18.1–23.8		38.71	35.3–42.1		30.65	27.4–33.9		89.52	87.4–91.7	
Professional formation	25.71	22.6–28.8		39.29	35.9–42.7		47.86	44.3–51.4		22.86	19.9–25.8		35.00	31.7–38.3		45.00	41.5–48.5		25.71	22.6–28.8		83.57	81.0–86.2	
Complete university	22.88	19.9–25.8		55.19	51.7–58.7		47.64	44.1–51.1		24.06	21.1–27.1		38.92	35.5–42.3		58.49	55.0–62.0		30.19	27.0–33.4		88.92	86.7–91.1	
**Family members (*N* = 820)**			0.7388			0.164			0.0003 ***			<0.0001 ***			0.6579			0.859			0.3438			0.0163 *
≤3	5.61	4.1–7.5		13.17	10.9–15.5		10.37	8.3–12.5		3.41	2.2–4.7		8.05	6.2–9.9		12.93	10.6–15.2		6.71	5.0–8.4		21.34	18.5–24.1	
4–6	16.10	13.6–19.0		34.76	31.5–38.0		30.61	27.5–33.8		19.02	16.3–21.7		22.56	19.7–25.4		35.24	32.0–38.5		21.10	18.3–23.9		21.10	56.6–63.4	
≥7	1.71	0.9–2.9		2.44	1.4–3.5		2.20	1.2–3.2		2.56	1.5–3.6		2.44	1.4–3.5		3.29	2.1–4.5		1.71	0.8–2.6		6.22	4.6–7.9	
**Viewing TV during mealtimes (*N* = 820)**			0.4672			<0.0001 ***			0.0003 ***			0.1811			0.0001 ***			<0.0001 ***			0.2881			0.4256
Always	25.07	22.1–28.0		41.51	38.1–44.9		36.29	33.0–39.6		22.19	19.3–25.0		25.85	22.9–28.8		43.86	40.5–47.3		27.15	24.1–30.2		84.07	81.6–86.6	
Sometimes	20.25	17.5–23.0		57.06	53.7–60.4		44.79	41.4–48.2		25.77	22.8–28.8		36.20	32.9–39.5		53.37	50.0–56.8		29.45	26.3–32.6		29.45	86.1–90.5	
Never	22.99	21.0–25.9		58.76	55.4–62.1		51.82	48.4–55.2		28.47	25.4–31.6		41.24	37.9–44.6		60.95	57.6–64.3		32.85	29.6–36.1		91.97	90.1–93.8	

Chi-square test. Data are shown as percent of adolescents and respective 95% confidence intervals (95%CI) for the outcome weighted for these variables. * *p* < 0.05, ** *p* < 0.001, *** *p* < 0.0001. *N* = 820.

**Table 4:** Analysis of mean dietary intake among adolescents during COVID-19 confinement by country.

	Legumes		Fried food		Vegetables		Sweet Food		Fruits		Sugar-Sweetened Beverages		Processed Meat		Fast Food	
	*p* Value		*p* Value		*p* Value		*p* Value		*p* Value		*p* Value		*p* Value		*p* Value	
Spain vs. Italy	0.0065	**	0.001	***	0.6246	ns	<0.0001	***	0.0621	ns	0.4204	ns	>0.9999	ns	0.745	ns
Spain vs. Brazil	<0.0001	***	0.0048	**	0.0882	ns	<0.0001	***	<0.0001	***	0.3736	ns	0.0513	ns	0.177	ns
Spain vs. Colombia	0.999	ns	0.2521	ns	0.0015	**	0.7525	ns	<0.0001	***	<0.0001	***	0.6983	ns	0.2613	ns
Spain vs. Chile	<0.0001	***	0.1303	ns	0.0073	**	0.2396	ns	0.0004	***	0.0023	**	0.0734	ns	0.2284	ns
Italy vs. Brazil	<0.0001	***	>0.9999	ns	0.001	***	0.7779	ns	0.0007	***	0.9989	ns	0.0513	ns	0.7745	ns
Italy vs. Colombia	0.002	**	<0.0001	***	<0.0001	***	<0.0001	***	<0.0001	***	0.0004	***	0.606	ns	0.9124	ns
Italy vs. Chile	0.8563	ns	0.3835	ns	0.283	ns	<0.0001	***	0.6211	ns	0.2878	ns	0.0409	*	0.9137	ns
Brazil vs. Colombia	<0.0001	***	<0.0001	***	0.8752	ns	<0.0001	***	0.9641	ns	0.0062	**	0.0008	***	0.9959	ns
Brazil vs. Chile	<0.0001	***	0.5245	ns	<0.0001	***	<0.0001	***	0.0347	*	0.6008	ns	<0.0001	***	0.9914	ns
Colombia vs. Chile	<0.0001	***	<0.0001	***	<0.0001	***	0.9335	ns	0.0008	***	0.121	ns	0.7343	ns	>0.9999	ns

Data are means ± SEM. In the table, comparison between groups by paired two-way ANOVA. * *p* < 0.05, ** *p* < 0.001, *** *p* < 0.0001, ns: non-significant.

## Supplementary Materials

The following are available online at https://www.mdpi.com/2072-6643/12/6/1807/s1, Table S1: National responses to the COVID-19 pandemic, Table S2: Percentage of adolescent participating in this survey classified by countries and regions, Figure S1: Flow chart of participants of the study.
