# Supplementary material for: Covid-19 Confinement and Changes of Adolescent’s Dietary Trends in Italy, Spain, Chile, Colombia and Brazil
Source: Nutrients. 2020 Jun 17;12(6):1807. doi: 10.3390/nu12061807 (PMC7353171; doi:10.3390/nu12061807)
Supplement: Supplementary file 1 [file nutrients-12-01807-s001.pdf]

Supplementary Table S1. National responses to the COVID-19 pandemic.

		COVID-19 pandemic lockdowns		
**Countries and territories**	**Place**	**Start date**	**Level**	**Containment measures**
Italy		08/03/2020	National	Closed their borders and total quarantine were decreed[1]
Spain		14/03/2020	National	At first, the central government retains all powers and all police are under the control of the Interior Ministry. Many nonessential activities are forbidden, including large gatherings, restaurants, museums. Finally, stay-at-home orders was imposed[2]
Brazil	Santa Catarina<br>São Paulo	17/03/2020<br>24/03/2020	State	Closed their borders, prohibition of interstate travel, restriction of items purchased in the markets. Social distancing plan in some states were decreed and schools were closed[3]
Colombia		25/03/2020	National	Closed their borders, and stay-at-home orders were imposed[4]
Chile	Santiago<br>Independencia<br>Providencia<br>Ñuñoa<br>Las Condes<br>Vitacura<br>Lo Barnechea<br>Padre las Casas<br>Temuco<br>Punta Arenas	26/03/2020<br>28/03/2020<br>01/04/2020	State	Mandatory quarantine by states and nationwide nightly curfew were decreed. Schools were closed[5]

[1] "Il governno firma il decreto coronavirus: l'Italia divisa en 3 zonas". la Repubblica; [2] "Pedro Sánchez anuncia el estado de alarma para frenar el coronavirus 24 horas antes de aprobarlo". ELMUNDO; [3] "Doria rebate Bolsonaro: 'estamos fazendo o que ele não faz, liderar'". noticias.uol.com.br; [4] "Colombia announces lockdown as coronavirus cases surge". aa.com.tr.; [5] "Chile announces nationwide nightly curfew, coronavirus cases hit 632". Nationalpost.

Supplementary Table S2. Percentage of adolescent participating in this survey classified by countries and regions.

Country and region	%
**Spain**	**18.54**
Madrid	75.00
Andalucía	9.21
Múrcia	5.92
Castilla y León	3.29
Islas Baleares	1.97
Navarra	1.97
Castilla La Mancha	1.32
Cantabria	0.66
Canarias	0.66
**Italy**	**19.63**
Vicenza	39.55
Verona	21.47
Rovigo	15.82
Padova	11.86
Lombardía, Mantua	3.95
Venezia	3.95
Lombardía, Lodi	1.69
Belluno	1.69
**Brazil**	**14.02**
Rio de Janiero	95.65
São Paulo	3.48
Pernambuco	0.87
**Colombia**	**19.63**
Bolívar	70.19
Atlántico	8.70
Antioquia	6.21
Bogotá	5.59
Sucre	3.73
Tolima	2.48
Arauco	1.86
Santander	0.62
Córdoba	0.62
**Chile**	**26.22**
Concepción	63.26
Biobío	20.93
Santiago	8.84
Diguillín	2.79
Arauco	1.40
Llanquihue	0.93
Palena	0.47
Antofagasta	0.47
Arica	0.47
Punilla	0.47

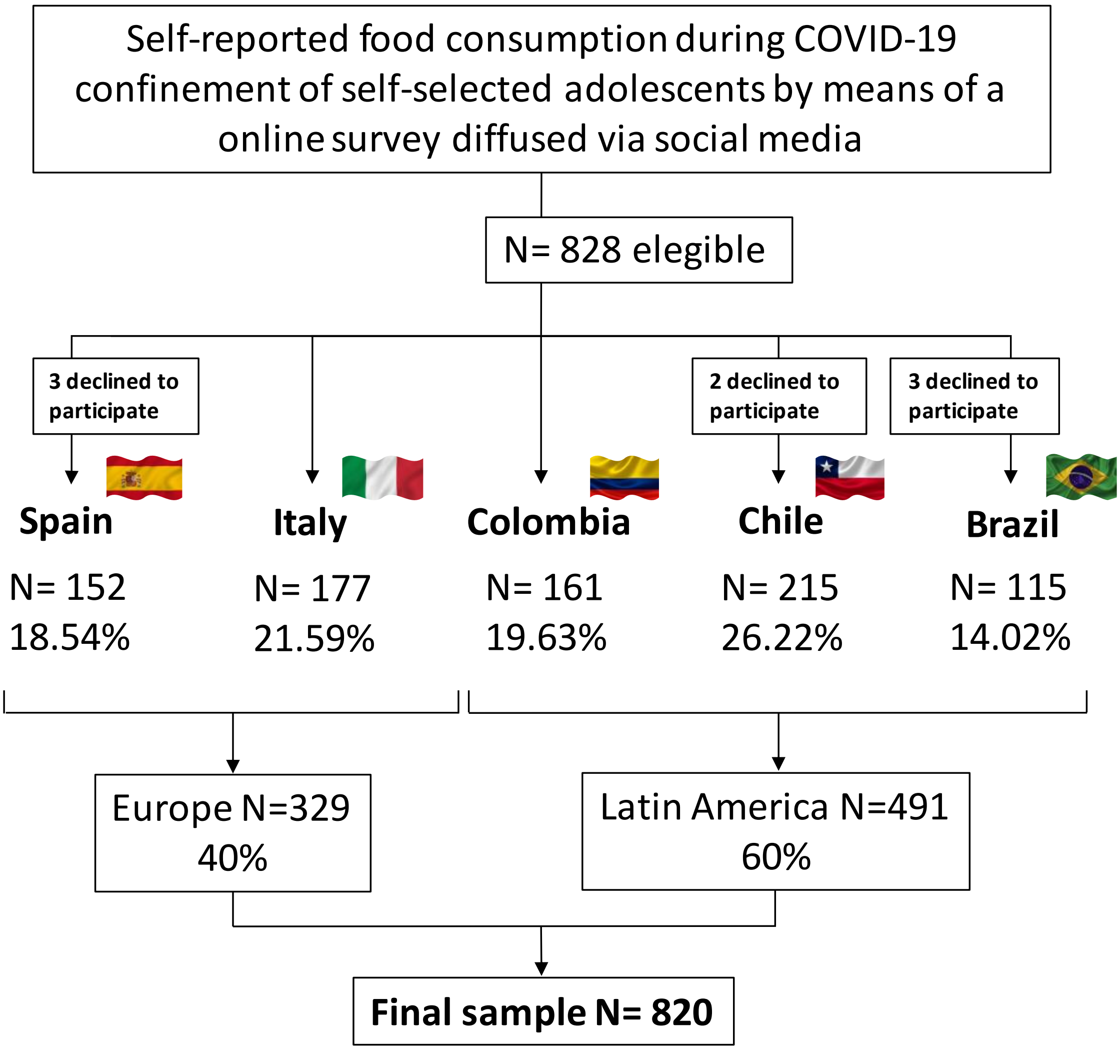

Supplementary Figure S1. Flow chart of participants of the study.
